# Oxidative Stress, MicroRNAs, and Long Non-Coding RNAs in Osteoarthritis Pathogenesis: Cross-Talk and Molecular Mechanisms Involved

**DOI:** 10.3390/ijms26136428

**Published:** 2025-07-03

**Authors:** Teresa Iantomasi, Cinzia Aurilia, Simone Donati, Irene Falsetti, Gaia Palmini, Roberto Carossino, Roberto Zonefrati, Francesco Ranaldi, Maria Luisa Brandi

**Affiliations:** 1 Department of Experimental and Clinical Biomedical Sciences “Mario Serio”, University of Florence, 50139 Florence, Italy; cinzia.aurilia@unifi.it (C.A.); simone.donati@unifi.it (S.D.); irene.falsetti@unifi.it (I.F.); roberto.carossino@unifi.it (R.C.); francesco.ranaldi@unifi.it (F.R.); 2 FirmoLab, Fondazione F.I.R.M.O. Onlus and Stabilimento Chimico Farmaceutico Militare (SCFM), 50141 Florence, Italy; gaia@fondazionefirmo.com (G.P.); zonefrati@gmail.com (R.Z.); marialuisa@marialuisabrandi.it (M.L.B.)

**Keywords:** oxidative stress, osteoarthritis, miRNAs, lncRNAs, cartilage degradation

## Abstract

Osteoarthritis (OA) is the most common degenerative joint disease, characterized by articular cartilage degradation, synovial inflammation, and ligament lesions. Non-coding RNAs (ncRNAs) do not encode any protein products and play a fundamental role in regulating gene expression in several physiological processes, such as in the regulation of cartilage homeostasis. When deregulated, they affect the expression of genes involved in cartilage degradation and synovial inflammation, contributing to the onset and progression of OA. Oxidative stress is also involved in the pathogenesis of OA by contributing to the inflammatory response, degradation of the extracellular matrix, and induction of chondrocyte apoptosis. Studies in the literature show a reciprocal relationship between the altered expression of a number of ncRNAs, including microRNAs (miRNAs) and long non-coding RNAs (lncRNAs), and oxidative stress. The aim of this review is to highlight the role of oxidative stress, miRNAs, and lncRNAs and their cross-talk in OA in order to understand the main molecular mechanisms involved and to identify possible targets that may be useful for the identification and development of new diagnostic and therapeutic approaches for this disease.

## 1. Introduction

Osteoarthritis (OA) is a chronic and degenerative disease that affects the joints, causing structural disorders to cartilage and surrounding tissues. It mainly affects the elderly and alters their quality of life, being one of the main causes of disability [1]. The joints that are prevalently affected by OA are the hips, ankles, knees, hands, feet, cervical, and lumbar spine, and temporal and mandibular joints [2,3]. The symptoms of OA develop slowly and get worse over time. Joint pain, stiffness, and loss of flexibility, as well as bone spurs and swelling, result from inflammation of the synovium, the soft tissue surrounding the joint [4]. Loss of articular cartilage and subchondral bone, tissue hypertrophy, laxity of tendons and ligaments, and hypervascularization of the synovium occur when the pathology is in an advanced state [5]. Cartilage coats and lubricates bone surfaces at joints and absorbs stresses during movement, and chondrocytes in cartilage regulate the balance of protein metabolism, which is shifted toward catabolism in OA. This induces extracellular matrix (ECM) degradation, resulting in cartilage damage [6,7]. In OA, cartilage integrity is also affected by changes in the levels of lubricin and hyaluronic acid, components of the synovial fluid produced by the synovium to maintain proper cartilage and chondrocyte function [8]. Although OA is a condition due to mechanical stresses, it is also caused by the infiltration of inflammatory cells into the synovial membrane and the consequent production of pro-inflammatory cytokines [9,10]. Risk factors for OA are diverse and include genetics, aging, obesity, gender, joint injury, and some metabolic diseases. In addition, the risk of OA is increased in individuals with a genetic predisposition and in older individuals in whom the presence of age-related inflammation leads to changes in ECM components and articular cartilage [11,12,13]. Moreover, considering that OA is predominantly associated with menopause, women are more likely to develop this pathology compared to men [14]. Indeed, in chondrocytes, the lack of estrogen inhibits proteoglycan production and stimulates the production of reactive oxygen species (ROS), as well as activating the enzyme inducible nitric oxide synthase (iNOS), and the transcription factor nuclear factor-kappa B (NF-κB) [15,16]. It is well known that oxidative stress plays an important role in the onset and progression of OA by inhibiting new cartilage synthesis and the migratory and proliferative capacity of chondrocytes [17]. In addition, activation of NF-κB is critical in OA and upregulates the expression of inflammatory mediators, including iNOS and cyclooxygenase-2 (COX-2), and proteinases involved in cartilage degradation [18].

Obesity is one of the greatest risks for OA; in fact, increased body weight increases stress on weight-bearing joints such as the hips and knees, causing ligament damage. Moreover, adipose tissue produces cytokines that can cause inflammation in and around the joints, leading to failure of ECM components and cartilage degeneration [19,20].

Injury to the joint, along with the resulting increase in inflammatory cytokines in the synovial fluid, are implicated in the development of OA. In fact, joint trauma in the form of fractures, cartilage damage, or ligament tears, even those that occurred many years ago and appear to have healed, are a significant risk factor for OA. People who suffer a joint injury have a much higher risk of developing OA than those who are not injured [21,22].

Some metabolic diseases, such as hypertension, type II diabetes, and hemochromatosis, are risk factors for OA. In particular, individuals with hypertension appear to be more susceptible to OA, as vasoconstriction and subchondral ischemia may trigger cartilage degradation [23,24]. The link between type II diabetes and OA has been demonstrated by the fact that chondrocytes isolated from patients with OA accumulate significantly more glucose than those isolated from healthy donors. This results in excessive production of ROS and the overexpression of matrix-degrading enzymes such as metalloproteinases (MMPs), as well as cytokine production. Consequently, signaling pathways involved in cartilage degradation and cell apoptosis are activated [25,26]. Conditions characterized by an iron overload, such as hemochromatosis, thalassemia, hemophilia, but also aging and estrogen deficiency, are related to OA [27,28]. In chondrocytes, large amounts of iron upregulate the expression of MMP-3 and MMP13, leading to cartilage damage, and promoting apoptosis by inducing oxidative stress [29,30].

Several pro-inflammatory cytokines, such as interleukin (IL)-1β, tumor necrosis factor (TNF)α, and IL-6, secreted by immune cells influence chondrocyte metabolism and are involved in the development of OA [31,32]. They upregulate the expression of genes that promote the production of nitric oxide (NO) and prostaglandin E2 (PGE2), which increase articular inflammation and injury through activation of MMP expression, chondrocyte apoptosis, and inhibition of collagen and proteoglycan synthesis [33]. Specifically, IL-1β upregulates ROS production and downregulates ROS scavenging enzymes, accelerating cartilage degradation in OA [34]. ROS and cytokines can activate the Janus kinase signal transducer and activator of transcription (JAK/STAT) and Wnt/β-catenin signaling pathways. This increases the production of inflammatory mediators and induces oxidative stress, which leads to ECM degradation and synovial inflammation in chondrocytes [35,36,37]. Indeed, in OA, the Wnt/β-catenin pathway also increases the expression of A disintegrin and metalloproteinase with thrombospondin motifs (ADAMTS)4 and ADAMTS5, highly expressed in OA cartilage [37,38].

As evidenced in the above-mentioned paragraphs, OA is a complex, multifactorial disease. Epigenetics plays a crucial role in the regulation of various genes [39] and represents a reversible mechanism for phenotypic inheritance of transcription control, independently of alterations to the DNA nucleotide sequence. It includes tissue-specific regulatory actions of gene expression, such as DNA methylation, modifications of histones, and gene silencing via non-coding RNAs (ncRNAs) [40]. ncRNAs are RNAs that are transcribed from DNA but not translated into proteins. They are no less important than protein-coding mRNAs and are involved in the regulation of gene expression in several fundamental cellular processes [41]. In particular, they play an important role in cartilage homeostasis, which is essential for joint function, by influencing chondrocyte differentiation and proliferation and ECM biosynthesis [42]. When deregulated, they are one of the epigenetic factors that play an important role in contributing to the onset and progression of OA through synovial inflammation; ECM degradation; and chondrocyte hypertrophy, senescence, and apoptosis [43,44]. Then, epigenetics has a significant impact on the development and preservation of joints in OA, compromising the capacity of articular chondrocytes to sustain physiological activities [45].

Oxidative stress is an early event involved in the onset of OA and contributes to its progression through its reciprocal interaction with MMPs and cytokines. In this context, there are also ncRNAs whose expression can be altered by oxidative stress; for example, several ncRNAs can affect the intracellular redox state by increasing or inhibiting ROS production [46,47]. Among ncRNAs, microRNAs (miRNAs) and long non-coding RNAs (lncRNAs) play a central role in regulating gene expression and modulating the cellular response to pathophysiological stresses, such as oxidative stress [48,49]. In recent years, several investigations have highlighted the importance of epigenetics OA. It is becoming increasingly evident that the interplay between oxidative stress and epigenetic factors, along with the resulting alterations in gene expression, could influence the onset and progression of OA by regulating the transition of quiescent articular chondrocytes toward a hypertrophic phenotype. Then, considering all this and that cytokines and MMPs are well-established key mediators of cartilage degradation, this review will focus on the role of oxidative stress and altered expression of miRNAs and lncRNAs in the pathogenesis of osteoarthritis (OA), highlighting the cross-talk between them.

The aim is to identify molecular targets and pathways that may be useful in identifying new therapeutic approaches to attenuate the progression of OA and/or alleviate its symptoms.

**Figure 1:**
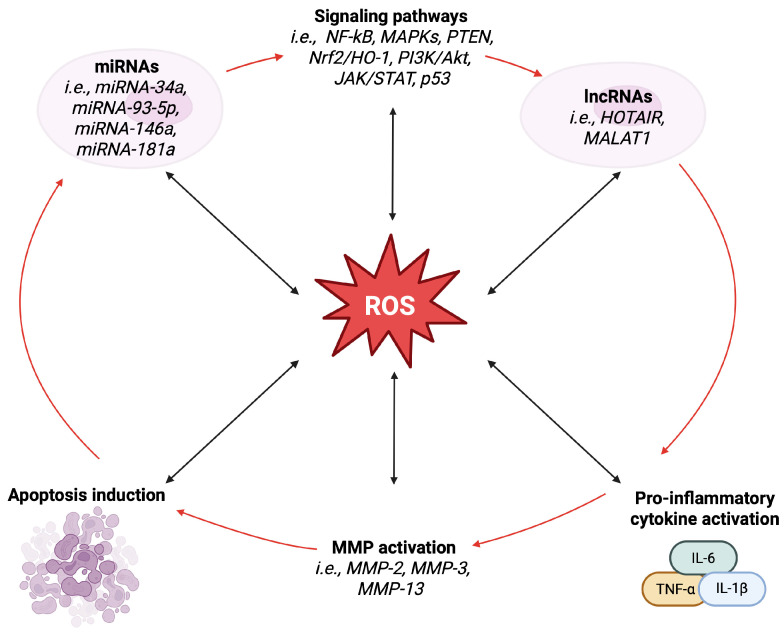
Intricate connections among MMPs, cytokines, miRNAs, lncRNAs, and oxidative stress. Abbreviations: ROS, reactive oxygen species; NF-κB, nuclear factor kappa B; MAPKs, mitogen-activated protein kinases; TNFα, tumor necrosis factor α; MMP, metalloproteinase; IL-1β, interleukin-1β; IL-6, interleukin-6; PTEN, phosphatase and tensin homologue of chromosome 10 PI3K/Akt, phosphatidylinositol-3-kinase/protein kinase B; Nrf2/HO-1, nuclear factor erythroid 2-related factor 2/heme oxygenase-1; JAK/STAT, Janus kinase/signal transducer and activator of transcription. Image created by BioRender (https://app.biorender.com, accessed on 30 June 2025).

Figure 1 illustrates the complex network of relationships between MMPs, cytokines, miRNAs, and lncRNAs, with oxidative stress playing a key role.

## 2. Oxidative Stress and Osteoarthritis

Under physiological conditions, chondrocytes, metabolically active cells involved in the synthesis of ECM components such as collagen, glycoproteins, proteoglycans, and hyaluronan, live in an avascular environment. As a result, they are adapted to anaerobic metabolism and receive the oxygen they need for their biological functions from the synovial fluid [50].

ROS production, mainly due to activation of NADPH oxidase (NOX), a resting membrane enzyme complex, is very low in chondrocytes and plays an important role in maintaining cartilage homeostasis and chondrocyte function by modulating apoptotic processes and gene expression, as well as ECM metabolism and cytokine synthesis [17,51,52]. Mechanical stress; changes in the partial pressure of oxygen in the synovial fluid; and other factors, such as pro-inflammatory cytokines, present in the chondrocyte environment can alter chondrocyte metabolism and induce excessive increases in ROS, particularly NO and superoxide anion (O_2_^●−^), from which other radicals are generated [53,54,55]. The increase in ROS, if not counterbalanced by an equal increase in antioxidant systems, creates a state of oxidative stress that induces changes in the biological functions of proteins and affects signaling pathways. This is a critical factor in the development of OA, as high levels of ROS and low levels of antioxidants have been found in human OA joints, cartilage, and chondrocytes, causing inflammation and chondrocyte apoptosis [56,57]. In particular, the antioxidant properties of the glutathione (GSH) system and the activity of intracellular redox state-related enzymes, such as superoxide dismutase (SOD) and catalase, are reduced in OA [58,59]. On the contrary, pro-inflammatory mediators increase in the synovial fluid of joints with OA and induce ROS production, which in turn stimulates the inflammatory response by regulating the expression of cytokines and interleukins through the activation of c-Jun N-terminal kinases (JNK) in chondrocytes [17,60]. Age, obesity, and vitamin D levels may be partly responsible for the development of OA through changes in the redox state of the synovial fluid [61]. In particular, vitamin D deficiency is strongly associated with oxidative stress and increased MMP activity in patients with knee OA [62]. It also causes age-related OA, which can be prevented by administering exogenous 11,25(OH)_2_D_3_, a metabolite of vitamin D that activates sirtuin-1 (Sirt1), a class III protein deacetylase that reduces oxidative stress [63,64,65]. Hyaluronic acid injections with vitamin D reduce oxidative stress in synovial fluid, decrease knee pain, and improve knee function in OA patients with vitamin D insufficiency [61]. ROS upregulate the activity and expression of MMPs, which in turn can induce oxidative stress and reduce cartilage synthesis by limiting chondrocyte stimulation by growth factors [66,67,68,69]. This prevents adequate repair of the injury by reducing the migration and proliferative capacity of chondrocyte precursors within the damaged areas [70,71].

An association between oxidative stress and epigenetic DNA methylation changes in OA has been reported in the literature. The upregulation of DNA methyltransferases can suppress peroxisome proliferator-activated receptor gamma and cause the methylation of the promoters of iNOs and COX-2, thereby promoting oxidative stress in OA [72,73] An increase in partially methylated CpG sites in the SOD-2 promoter, as well as upregulation of the non-histone chromatin structural protein, high mobility group A1, may be involved in OA progression by exacerbating oxidative stress and the inflammatory response [59,74,75].

### 2.1. MAPKs, NF-κB, and PI3K/Akt Pathways

High ROS accumulation activates mitogen-activated protein kinases (MAPKs) and NF-κB, which regulate genes involved in cartilage metabolism and inflammation; and it also causes chondrocyte senescence by activating Krüppel-like transcription factor 10 (KLF10), involved in several biological processes, including proliferation, apoptosis, and differentiation [76,77,78]. NO is critical for chondrocyte apoptosis through activation of caspase-3 and caspase-9, and cross-talk between iNOS and COX-2, mediated by activation of p38-MAPK and MAPK kinases (MEK1/2) [79,80]. In addition, oxidative stress promotes chondrocyte death and premature senescence through the overexpression of caveolin-1 due to the activation of p38-MAPK, NF-κB, and COX-2 expression [81,82]. In the mouse temporomandibular joint (TMJ), mechanical stress in the synovial fluid induces ROS production that may contribute to synovial inflammation and OA by activating MAPKs, particularly extracellular signal-regulated kinases (ERK)1/2. This leads to overexpression of the neutrophil chemoattractant CXCL15/Lungkine in fibroblast-like synoviocytes, with consequent recruitment of neutrophils into the joint and amplification of inflammatory processes [83]. In contrast, the inhibition of both ROS-stimulated NF-κB and MAPKs pathways delays the progression of TMJ-OA in rats and reduces ECM degradation and chondrocyte apoptosis [84]. The balance between MAPKs and phosphatidylinositol 3-kinase/protein kinase B (PI3K/Akt) is a very important factor in the pathogenesis of OA, and oxidative stress regulates these signaling pathways a different way in chondrocytes [77]. In chondrocytes, excessive ROS production is also linked to the inhibition of PI3K/Akt activity by overexpressing phosphatase and tensin homologue of chromosome 10 (PTEN), a negative regulator of this signaling pathway [85,86]. Hydrogen peroxide (H_2_O_2_) induces apoptosis, degrades collagen type II (Col2), and activates autophagy and expression of MMP3 and MMP13 by inhibiting the classical PI3K/Akt/mammalian target of rapamycin (mTOR) signaling pathway in human chondrocytes [87]. Megakaryoblastic leukemia 1, a transcriptional coactivator involved in several inflammatory processes, is overexpressed in H_2_O_2_-treated chondrocytes from OA rats, and its inhibition attenuates inflammation and cell apoptosis through Twist-related protein 1-mediated activation of the PI3K/AKT signaling pathway [88]. In fact, PI3k/Akt activation also reverses the induction of apoptosis and matrix degradation in primary rat chondrocytes treated with sodium nitroprusside, which promotes ROS production and mimics the progression of OA [89]. In chondrocytes, ROS inhibit the production of proteoglycans by activating ERKs and inhibiting PI3K/Akt [76], as well as reducing ATP levels [90]. However, some studies show that H_2_O_2_ treatment induces apoptosis by activating MAPK and PI3K/Akt in chondrocytes [91,92]. Withaferin A, a steroidal lactone that represents the principal constituent of Withania somnifera, also known as Indian ginseng, causes oxidative stress and the consequent loss of collagen and upregulation of COX-2 expression by activating PI3K/Akt, p38 MAPK, and JNK pathways in rabbit articular chondrocytes [93]. In addition, in human OA chondrocytes, p38 MAPK and JNK are also involved in the upregulation of MMP-13 and ADAMTS5 expression [78]. The latter mediates aggrecan cleavage and is the main aggrecanase associated with OA pathogenesis, considering that, when it is overexpressed, it represents a crucial risk factor in the degeneration of joints [94].

Cytokines promote cartilage destruction through the production of ROS, the activation of NF-κB, and the subsequent overexpression of MMPs [95,96]. In fact, stimulation of chondrocytes with IL-1β, a key cytokine released during the onset of OA and used to mimic OA in vitro, activates NOX and induces ROS production, oxidative stress, apoptosis, and ECM degradation in mouse OA chondrocytes [97]. IL-1β also increases in human chondrocytes’ MMP-1, MMP-13, and ADAMTS4 levels by activating NOX4, an isoform of NOX whose deficiency reduces OA severity in a mouse model of OA [98,99]. However, in addition to NOX activation, inhibition of the transcriptional factor forkhead O (FOXO), which regulates the expression of antioxidant enzymes, is also involved in the increased expression of MMP13 and decreased expression of aggrecan [100].

IL-1β-induced oxidative stress activates both PI3K/Akt and/or NF-κB/MAPKs and promotes ECM degradation, apoptosis, and inflammatory response in human chondrosarcoma SW1353 cell line and primary mouse and rat chondrocytes [101,102,103]. The activation of NF-κB associated with the overexpression of hypoxia-inducible factor (HIF)2α, an important catabolic mediator regulated by NF-κB in OA progression [104,105], is also involved in IL-1β-mediated downregulation of the expression of cartilage-specific genes, such as SRY-box transcription factor 9 (SOX9) and collagen type II alpha 1 chain (Col2a1) [104,106]. In vitro and in vivo studies show that compounds that reduce the levels of ROS help to restore the homeostasis of the ECM and alleviate chondrocyte senescence and cartilage degeneration in OA by inhibiting the ROS-stimulated NF-κB signaling pathway [106,107,108].

**Figure 2:**
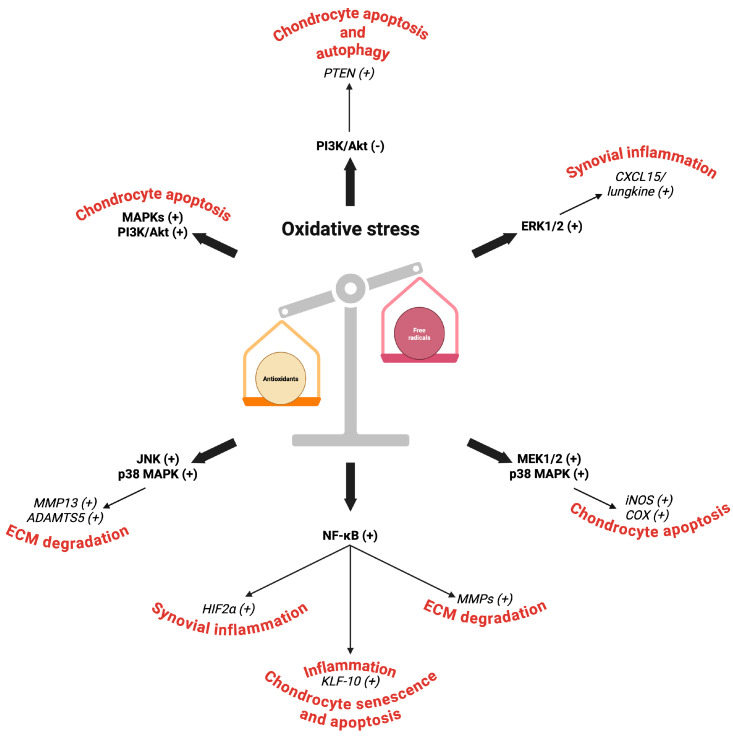
Principal molecular mechanisms involved in the MAPK, NF-κB, and PI3K/Akt signaling pathways activated by oxidative stress in OA. Abbreviations: PI3K/Akt, phosphatidylinositol-3-kinase/protein kinase B; MAPKs, mitogen-activated protein kinases; NF-κB, nuclear factor kappa B; ERK1/2, extracellular signal regulated kinases 1 and 2; JNK, c-Jun N-terminal kinase; MEK1/2, MAPK kinases; ADAMTS5, disintegrin and metalloproteinase with thrombospondin motifs 5; MMP, metalloproteinase; HIF2α, hypoxia-inducible factor 2 alpha; iNOS, inducible nitric oxide synthase; KLF-10, Krüppel-like factor 10; COX, cyclooxygenase; ECM, extracellular matrix. (−) Downregulated. (+) Upregulated. Image created by BioRender (https://app.biorender.com, accessed on 30 June 2025).

Figure 2 summarizes the principal molecular mechanisms and the effects in OA of oxidative stress-activated MAPKs, NF-κB, and PI3K/Akt signaling pathways.

### 2.2. Nrf2/OH-1 Signaling Pathway

ROS induce cartilage damage by downregulating the expression of the transcriptional factor, nuclear factor erythroid-2 related factor (Nrf2), with the function of regulating the cellular oxidative stress response and the HO-1 (heme/oxygenase 1) gene, which is involved in the regulation of cellular functions such as proliferation and inflammation [109,110]. In animal models of OA and IL-1β-stimulated chondrocytes, inhibition of Nrf2/HO-1, associated with NF-κB activation, due to oxidative stress may be responsible for chondrocyte apoptosis and OA progression [111,112]. Murine chondrocytes stimulated with tert-butyl hydroperoxide (tBHP) show increased levels of ROS, decreased nuclear translocation of Nrf2, and inhibition of Nrf2/HO-1, resulting in mitochondrial dysfunction and apoptosis [113,114]. For this, the activation of Nrf2 and the HO-1 signaling pathways is very important for preserving the chondrocyte homeostasis and cartilage integrity [110,115,116] and alleviating OA in rats [117]. In fact, it has been shown that enhancement of the Nrf2/HO-1 signaling pathway and/or inhibition of PI3K/AKT/NF-κB and MAPK pathways promotes the antioxidant capacity of chondrocytes, protects cartilage from degradation, and alleviates OA in rats [85,97,118,119,120].

### 2.3. Ferroptosis

Iron is a trace element that regulates several biological processes in humans. Its excess causes oxidative stress, an increase in chondrocyte apoptosis, and the overexpression of metalloproteinases, inducing cartilage damage that can be alleviated by the activation of Nrf2/HO-1 [121,122]. Ferroptosis, a novel form of iron-regulated cell death characterized by high levels of ROS and lipid peroxidation, contributes to the pathogenesis of OA, as chondrocytes undergo ferroptosis under conditions of inflammation and iron overload. Iron has been shown to accumulate in cartilage and synovial fluid, while markers of the peroxidation defense system, such as glutathione peroxidase 4 (GPX4) and GSH levels, decrease as OA progresses [123]. Ferroptosis increases oxidative stress and the expression of NOX1, HIF2α, and MMPs, while it inhibits the expression of Col2 in chondrocytes [124,125]. In fact, the oxidative stress/NF-κB/HIF2α signaling pathway is involved in the chondrocytes’ ferroptosis and cartilage degeneration by increasing the expression of MMP3 and MMP13 [124]. The oxidative stress-related HIF-1α pathway also plays a key role in iron homeostasis and OA, and gut microbiota attenuate ferroptosis-dependent OA by specifically reducing oxidative stress and HIF1α levels [126]. Under conditions of iron overload, ROS generation can downregulate Col2 expression and upregulate MMP expression also by inhibiting Nrf2/HO-1 and GPX4/cysteine/glutamate exchanger signaling pathways [127]. GPXs are involved in the antioxidant response and play a very important role in the pathogenesis of OA. However, high levels of GPX1 have been found in OA patients, and this upregulation can be explained by attributing to it a compensatory role against the oxidative stress that occurs during the onset and progression of OA [128].

### 2.4. Inflammasome

The inflammasome, a multimeric protein complex, is activated by numerous stimuli, including ROS and NF-κB, resulting in the maturation and secretion of IL-18 and IL-1β, which exacerbate the inflammatory response [129]. In addition to oxidative stress, increased levels of ILs and inflammasome components such as nucleotide-binding oligomerization-like receptor family pyrin domain-containing 3 (NLRP3), apoptosis-associated speck-like protein, caspase-1, and cleaved caspase-1 have been detected in OA cartilage biopsies and chondrocytes [130]. Furthermore, the expression of NLRP3 increases in association with the downregulation of the Nrf2/OH-1 pathway, and it is activated by H_2_O_2_ in OA rat chondrocytes [131,132]. This causes cleavage of gasdermin D, with consequent increases in pro-inflammatory mediators and MMPs [132]. During the onset and progression of OA synovitis, ROS contribute to the induction of the inflammatory phenotype (M1) of macrophages [133], whose high infiltration present in human OA synovium contributes to the progression of this pathology through the ROS/NLRP3 signaling pathway [134]. Among the adipokines that are implicated in the pathogenesis of OA, leptin activates the NLRP3 inflammasome through activation of NOX4 and subsequent ROS production [130].

## 3. miRNA and Osteoarthritis

miRNAs are small ncRNA molecules consisting of approximately 22 nucleotides of intragenic origin, mostly from introns, or intergenic origin. According to the canonical pathway, miRNAs derive from a primary transcript (pri-miRNA) that is cleaved by Drosha in the nucleus to yield the miRNA precursor (pre-miRNA). The pre-miRNA is transferred to the cytoplasm, where it is cleaved by Dicer to form a double-strand miRNA. This duplex is unwound, and the single strand is loaded on the RNA-induced silencing complex (RISC) [135]. By binding to the complementary sequence located in the 3’-untranslated regions of mRNA, the complex RISC-miRNA degrades mRNA or inhibits its translation, resulting in decreased gene expression [136]. Then, miRNAs affect biological processes and signaling pathways that contribute to the regulation of cell cycle, proliferation, differentiation, and death [137]. The contribution of miRNAs to OA pathology has been demonstrated using genetically engineered animal models. Specifically, it was shown that chondrocyte proliferation and differentiation are decreased and increased, respectively, in mice deficient in Dicer, an enzyme involved in miRNA biogenesis. The result is altered cartilage formation [138]. In addition, mice that lack miRNA-204/-211 are more likely to develop OA, and the disease is much more severe [139,140]. In fact, these two homologous miRNAs can maintain joint homeostasis and prevent OA development through the regulation of mesenchymal progenitor cell proliferation and differentiation [140]. Then, altered expression of specific miRNAs may contribute to the progression of OA by affecting chondrocyte function and increasing inflammation through the production of pro-inflammatory mediators such as cytokines and ROS. It may also promote cartilage degradation by increasing MMP expression [141]. Furthermore, miRNAs can be used as potential markers for OA, as a large number of differentially expressed miRNAs have been identified in the serum of OA patients.

An increase in the levels of miRNA-345-5p and miRNA-132-3p and a decrease in the levels of miRNA-127-3p and miRNA-382-5p were found in a rat model of OA and in human primary synovial cells stimulated with lipopolysaccharide (LPS) to mimic OA [142]. There is also an increase in the levels of both miRNA-122 and miRNA-451 in rat OA cartilage; however, in IL-1β-stimulated rat chondrocytes, the role of these two miRNAs is the opposite, as miRNA-122 reduces the effect of IL-1 β, whereas miRNA-451 increases it [143]. High levels of miRNA-203 and low levels of estrogen receptor α are detected in postmenopausal rats with consequent inflammation and cartilage destruction [144]. In contrast, downregulation of miRNA-4287, miRNA-142-3p, miRNA-18a-3p, and miRNA-130a induces an inflammatory response, cartilage damage, and chondrocyte apoptosis in a model of mouse OA [145,146,147,148].

### 3.1. miRNAs, Inflammation, and ECM Degradation

In human OA, the downregulation of several miRNAs contributes to the development and progression of OA by increasing the production of pro-inflammatory mediators and/or increasing the expression of proteases, such as ADAMTS4/5 and MMP3/13, involved in the ECM degradation. Reduced expression of miRNA-130a, miRNA-149, miRNA-373, and miRNA-24-3p increases the expression of cytokines such as TNFα, IL1β, IL-8, and IL-6, leading to inflammation [148,149,150,151]. In cartilage tissues of OA patients, decreased miR-149 levels are accompanied by an increase in the expression of vascular cell adhesion molecule-1 (VCAM-1) and phosphoprotein kinase B (pAkt), and this could promote inflammation and apoptosis through activation of the PI3K/Akt signaling pathway, as demonstrated in the cartilage of mice with OA [152]. In IL-1β-stimulated human chondrocyte cell line CHON-001, downregulation of miRNA-24-3p correlates with upregulation of B-cell leukemia 2-like 12, ADAMTS5, and MMP13, causing apoptosis, inflammation, and cartilage degradation [151]. miRNA-92a-3p and miRNA-497-5p are low expressed in human cartilage. In particular, a decrease in miRNA-92-3p and miR-497-5p causes an increase in metalloproteinases, such as ADAMTS4/5 and MMP13, and a decrease in the expression of cartilage matrix molecules via the activation of NF-κB/MAPKs and Wnt/β-catenin signaling pathways, respectively [153,154]. In addition, the expression of Wnt1 inducible signaling pathway protein 1 (WISP1), a product of the Wnt/β-catenin signaling pathway, is upregulated and associated with the downregulation of miRNA-128-3p in cartilage from OA patients. The WISP1 overexpression induces inhibition of chondrocyte proliferation, apoptosis, ECM degradation, and production of proinflammatory cytokines via the activation of the PI3K/Akt/NF-κB pathway [155]. The reduced expression of miRNA-382-3p is also involved in the development of OA by inducing inflammation and ECM degradation through the upregulation of connexin 43 (CX43) and the consequent increase in the expression of Toll-like receptor 4 (TLR4), myeloid differentiation primary response 88 (MyD88), and NF-κB [156]. miRNA-195, significantly overexpressed in human OA cartilage, causes cartilage destruction by downregulating parathyroid hormone-related protein (PTHrP) and increasing the expression of MMP-13 and Col X [157]. In OA synovial fluid, the reduced expression of miRNA-140 and miRNA-199 is associated with an increase in MMP3, leading to cartilage destruction [158]. The decrease in miRNA-548d-5p promotes excessive inflammatory cytokine release, altered ECM deposition, inhibition of cell growth, and enhanced apoptosis by increasing specificity protein 1 (SP1) expression in the IL-1β-stimulated human chondrocyte cell line C28/I2 [159]. On the contrary, increased levels of miRNA-381a-3p and miRNA-454 promote the release of IL-6 and IL-8 through the activation of NF-κB by decreasing the expression of the inhibitor kappa B-alpha (IkBα) and stanniocalcin-1, respectively [160,161]. Stimulation of primary human articular chondrocytes with TNFα or IL-1β induces increased levels of miRNA-760, which is significantly elevated in human OA tissues, along with decreased levels of heparin-binding EGF-like growth factor and increased cartilage degeneration [162]. Conversely, in mouse TNFα-treated chondrocytes, there is a decrease in miRNA-145, whose downregulation is involved in OA pathogenesis by activating MAPK kinase 4 [163]. miRNA-146a-5p and miRNA-122 are upregulated in human OA cartilage, leading to increased inflammation and cartilage degeneration [164,165]. In particular, the increase in miRNA-122 promotes ECM degradation by reducing the expression of Sirt1 [165].

**Figure 3:**
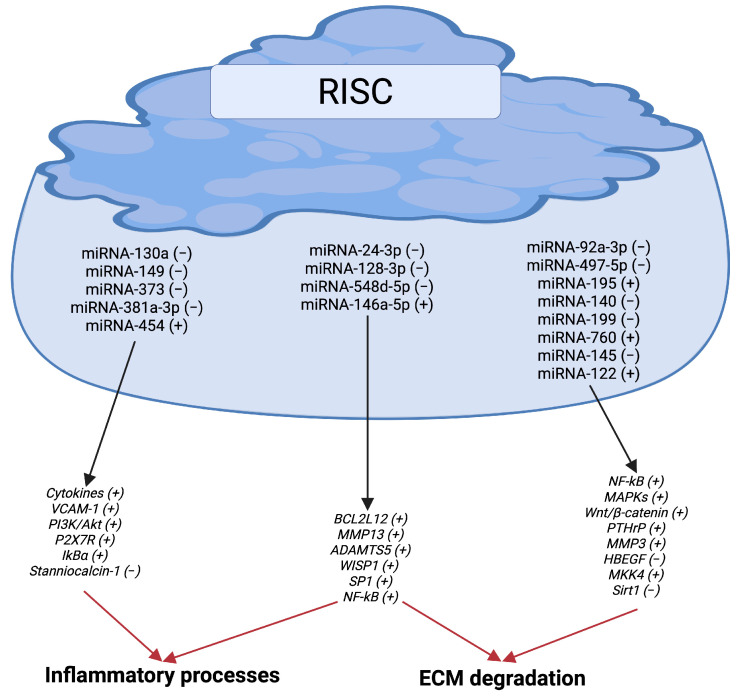
Principal microRNAs and targets involved in inflammation and cartilage destruction in OA. Abbreviations: NF-κB, nuclear factor kappa B; PI3K/Akt, phosphatidylinositol-3-kinase/protein kinase B; P2X7R, purinergic P2X7 receptor; VCAM-1, vascular cell adhesion molecule-1; IkBα, inhibitor kappa B-alpha; BCL2L12, B cell leukemia 2-like 12; MAPKs, mitogen-activated protein kinases; ECM, extracellular matrix; MMP, metalloproteinase; ADAMTS5, disintegrin and metalloproteinase with thrombospondin motifs 5; SP1, specificity protein 1; WISP1, Wnt-1 inducible signaling pathway protein 1; PTHrP, parathyroid hormone-related protein; MKK4, mitogen-activated protein kinase kinase 4; HBEGF, heparin-binding EGF-like growth factor; Sirt1, sirtuin-1. (−) Downregulated. (+) Upregulated. Image created by BioRender (https://app.biorender.com, accessed on 30 June 2025).

Figure 3 provides a summary of the microRNAs and their targets that are primarily involved in inflammation and cartilage destruction in OA.

### 3.2. miRNAs, Apoptosis, and Proliferation

During the progression of human OA, downregulation of miRNA-140-5p expression in cartilage progenitor/stem cells is associated with altered differentiation, increased apoptosis, and decreased proliferation through activation of Jagged1/Notch signaling [166]. Downregulation of miRNA-320a promotes inflammatory response and apoptosis and inhibits chondrocyte proliferation rate by increasing DAZ-associated protein 1 and MAPKs expression in an in vitro human chondrogenic HC-A cells OA model [167]. High levels of miRNA-146a contribute to the pathogenesis of human OA, and the application of mechanical pressure upregulates miRNA-146a expression, which causes apoptosis by increasing vascular endothelial growth factor (VEGF) and inhibiting Smad4 in chondrocytes [168]. Other microRNAs that affect apoptosis and proliferation also play a role in promoting cartilage degradation. In particular, in human OA tissues, downregulation of miRNA-379-5p increases the expression of Y-box binding protein 1 and also activates the PI3K/Akt pathway, resulting in decreased chondrocyte proliferation and expression of ECM-related proteins, collagen II, and aggrecan [169]. It has been shown that the downregulation of miRNA-99a expression, observed in the severe spine of OA patients, in the in vivo OA rat model, and in in vitro OA-like chondrocyte model, increases apoptosis and ECM degradation by overexpression of Frizzled 8, a positive regulator of Wnt/β-catenin pathways [170]. Moreover, in human OA cartilage and IL-1β-stimulated chondrocytes, the decrease in expression of miRNA-33b-3p correlates with the increase in expression of DNA methyltransferase 3A, reducing cell proliferation and causing apoptosis and cartilage ECM degradation [171].

Upregulation of miRNA-182-5p and miRNA-34a in human OA chondrocytes, in addition to inhibiting cell proliferation and increasing apoptosis, induces cartilage degradation by reducing the expression of fibroblast growth factor (FGF) 9, which regulates bone remodeling [172], and Sirt1 [173], respectively. Table 1 summarizes the effects and targets of the major differentially expressed miRNAs associated with the development and progression of human OA.

## 4. Cross-Talk Between Oxidative Stress and miRNAs in Osteoarthritis

**Figure 4:**
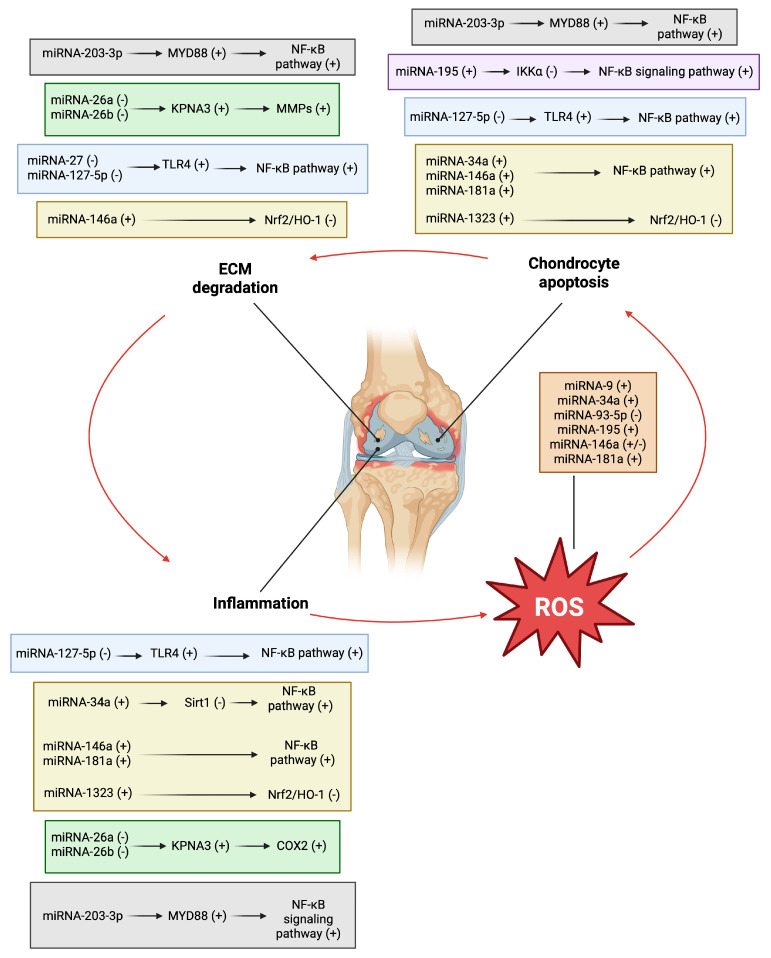
Principal signaling and effects of miRNAs related to oxidative stress. Abbreviations: ROS, reactive oxygen species; ECM, extracellular matrix; KPNA3, karyopherin subunit alpha 3; NF-κB, nuclear factor kappa B; MMP, metalloproteinase; MyD88, myeloid differentiation primary response 88; Nrf2/HO-1, nuclear factor erythroid 2-related factor 2/heme oxygenase-1; TLR4, Toll-like receptor 4; IKKα, inhibitory kappa B kinase α; Sirt1, sirtuin-1; COX2, cyclossigenase-2. (−) Downregulated. (+) Upregulated. Image created by BioRender (https://app.biorender.com, accessed on 30 June 2025).

Several miRNAs have been shown to regulate the redox state of cells under different pathological conditions by modulating signaling pathways involved in ROS and antioxidant production [174]. Various miRNAs are able to regulate the intracellular redox state by inhibiting the Nrf2 repressor, Kelch-like ECH-associated protein 1 [175,176]. In contrast, some miRNAs alter the intracellular redox state and induce oxidative stress by regulating genes involved in ROS production [177]. Oxidative stress may also be involved in altering miRNA expression levels by activating transcription factors such as p53, NF-κB, c-jun, FOXO, and HIF, or by causing epigenetic changes in miRNA gene methylation or post-translational histone modifications [178]. Thus, there is a close and reciprocal relationship between oxidative stress and miRNA expression, as demonstrated by a large body of experimental evidence [47,179]. The involvement of oxidative stress in the modulation of miRNA expression in OA has been demonstrated by stimulating human chondrocytes with H_2_O_2_, which increases ^●^O^2−^ levels, or with compounds capable of inducing oxidative stress. This suggests that a mutual interaction between miRNA and ROS production may also exist in this disease. miRNAs can induce oxidative stress, which can increase their own expression. In both cases, signaling pathways and effects are triggered that worsen the symptoms and quality of life of patients with OA by increasing the oxidative state (Figure 4).

### 4.1. Signaling Pathways Involved

In H_2_O_2_-treated human IL-1β-stimulated chondrocytes, there is a significant decrease in miRNA-93-5p and an increase in miRNA-449c-5p and miRNA-1207-5p, whereas miRNA-637 and miRNA-4763-3p do not change [180]. In particular, the oxidative stress-reduced levels of miRNA-93-5p, by activating MAPK kinase kinase (MAP3K8), increase the expression of pro-inflammatory cytokines, MMPs, and ADAMTS-4/5, and reduce the expression of Col2a1 and SOX9. In addition, low expression of microRNA-93-5p is capable of increasing oxidative stress by upregulating the expression of COX-2 and iNOS [180]. The oxidative stress induced by tBHP upregulates the expression of miRNA-34-5p, which causes joint degeneration, apoptosis, and inflammation by downregulating Sirt1 and upregulating p53 [181]. ROS-induced miRNA-9 and miRNA-195 expression may be involved in the development of OA by inhibiting Sirt1 and the expression of IKKα, an inhibitor of NF-κB [182,183]. In IL-1β-stimulated C28/I2 cells, decreased expression of mechanosensitive miRNA-222-3p promotes the production of ROS and pro-inflammatory mediators, ECM degradation, and apoptosis through increased expression of disintegrin and metalloproteinase domain-containing protein 10 (ADAM10), which is involved in cartilage degeneration [184]. Treatment of the murine chondrogenic ATDC5 cell line with LPS promotes oxidative stress, apoptosis, and inflammation by upregulating miRNA-486-5p and downregulating its target, Nrf1 [185]. Upregulation of miRNA-505-3p leads to increased production of ROS, apoptosis, overexpression of MMP3 and COX-2, downregulation of Col2, and mitochondrial dysfunction in OA by decreasing Sirt3 expression. This is a mitochondrial sirtuin that plays an important role in regulating oxidative stress by deacetylating compounds involved in mechanisms leading to ROS production or detoxification [186,187]. On the contrary, decreased expression of miRNA-26a and miRNA-26b detected in human OA cartilage and chondrocytes promotes cartilage degradation and ROS production by increasing MMP and COX-2 expression. This effect is mediated by upregulating the expression of karyopherin subunit alpha 3 (KPNA3), which is involved in p65 translocation to the nucleus and then in NF-κB activation [188,189].

Low expression of miRNA-485-3p in human OA cartilage promotes oxidative stress, apoptosis, inflammation, and ECM degradation by activating Notch2 and NF-κB [190]. Indeed, Notch signaling is involved in the pathogenesis of OA and can regulate oxidative stress by increasing ROS production or decreasing antioxidant systems. However, oxidative stress can also modulate the Notch signaling pathway [3,191]. The downregulation of miRNA-27 and miRNA-127-5p in human OA cartilage causes oxidative stress, increased expression of MMPs, and decreased activity of antioxidant enzymes such as SOD and GPX through the upregulation of TLR4, a molecule upstream of NF-κB activation [192,193]. The same effects are induced by the reduced expression of miRNA-24-3p, miRNA-197-3p, and miRNA-203-3p, but through the upregulation of cathepsin B, SOX5, and the MyD88/NF-κB pathway, respectively [194,195,196,197]. High levels of miRNA-181-5p and miRNA-375 cause oxidative stress and cellular damage by downregulating selenocysteine insertion sequence-binding protein 2 (SBP2), a regulator of antioxidant selenoprotein expression; the antioxidant enzyme GPX; and the JAK2/STAT3 signaling pathway [198,199]. The knockdown of miRNA-375 and subsequent activation of the JAK2/STAT3 pathway increases the ability of chondrocytes to counteract oxidative stress and maintain ECM homeostasis to prevent OA [199,200]. Adipokines, which, together with miRNAs and oxidative stress, are involved in synovitis and cartilage destruction in OA, induce oxidative stress by upregulating the expression of miRNA-34a, miRNA-146a, and miRNA181a, and subsequent activation of NF-κB in human OA synovial fibroblasts [201]. In addition, visfatin also increases miRNA-34a and miRNA-181a in human OA chondrocytes. These promote oxidative stress and apoptosis through NF-κB activation [202]. Treatment of human OA chondrocytes with H_2_O_2_ induces oxidative stress and promotes an increase in miRNA-34a and a decrease in miRNA-146a, demonstrating the responsiveness of these miRNAs to oxidative stress and confirming their role in the development and progression of OA [203].

### 4.2. Nrf2/HO-1 Signaling Pathway

There are conflicting data in the literature about the regulation of Nrf2 by miRNAs in OA. In particular, Nrf2 and SOD2 are targets of miRNA-146a; therefore, it is likely that the downregulation of miRNA-146a expression induced by H_2_O_2_ and associated with an increase in Nrf2 and SOD2 represents a form of defense to protect chondrocytes from the damaging effects of ROS [203,204,205]. However, other data show an upregulation of this miRNA in OA cartilage and in IL-1β-stimulated chondrocytes [206]. Even in rat OA chondrocytes, miRNA-146a is elevated and causes ROS production by inhibiting Nrf2 expression. In fact, melatonin, by downregulating miRNA-146a, increases Nrf2/HO-1 protein expression, and reduces ROS production and ECM degradation in rat OA chondrocytes [207]. Downregulation of Nrf2, associated with oxidative stress, inflammation, and apoptosis, is also correlated with an increase in miRNA-1323 in LPS-stimulated human OA chondrocytes [208]. miRNAs 34a, 146a, and 181a are also involved in the modulation of chondrocyte metabolism by hydrostatic pressure (HP), which regulates cartilage homeostasis in a force-, intensity-, and duration-dependent manner [187]. Their increase is involved in ROS production and upregulation of SOD and Nrf2 expression in human OA chondrocytes exposed to static continuous HP (10 MPa) for 3 h. This indicates a potential radical scavenging mechanism triggered by cells to counteract oxidative stress. The authors speculate that the upregulation of these miRNAs is due to the inhibition, of Sirt1 both at mRNA and protein levels [209]. Furthermore, these miRNAs have been shown to mediate oxidative stress, apoptosis, and upregulation of MMP13 and ADAMTS5 through the involvement of Wnt/β-catenin in HP (10 MPa)-treated OA chondrocytes [209].

### 4.3. Ferroptosis

In human and rat chondrocyte models of OA, miRNA-181b is upregulated and is involved in the development of ferroptosis by downregulating the solute carrier family 7 member 11 (SLC7A11), a ferroptosis protective protein [210]. The downregulated expression of miRNA-1 in human OA cartilage and IL-β1-stimulated human OA chondrocytes contributes to the increase in ferroptosis through upregulation of CX43 accompanied by enrichment of intracellular ROS and decrease in GPX4 and SLC7A11 [211].

Table 2 shows the targets and effects of the principal microRNA related to oxidative stress in OA.

## 5. lncRNAs and Osteoarthritis

lncRNAs are molecules of more than 200 nucleotides in length. They can be intergenic, antisense, exonic, intronic, or derived from 5′/3′ untranslated regions and adopt complex structures to facilitate their interactions with DNA, RNA, and proteins [212]. Due to their diverse biogenesis, lncRNAs are involved in a wide variety of biological processes, such as chromatin remodeling, transcriptional regulation, and the regulation of the integrity and function of the nuclear body [213,214,215]. Since a number of lncRNAs are exported to the cytoplasm, they play an important role in the regulation of mRNA stability, turnover, and translation, and in the modulation of post-translational modifications [216,217]. Finally, by binding to nucleic acids (RNA and DNA) or proteins, lncRNAs play an important role in the modulation of gene expression and/or regulation of signaling pathways, such as the NF-κB and p53 pathways [218,219]. For this, lncRNAs regulate various physiological processes, such as inflammation, cytokine expression, glucose and cholesterol metabolism, and cellular signal transduction [220,221,222,223]. lncRNAs can also affect the role of miRNAs, and the effect of lncRNAs on miRNA is twofold: they can sequester miRNAs, thereby inhibiting their function, or they can be precursors of miRNAs. However, the stability of lncRNAs can also be affected by interaction with specific miRNAs [224].

Under physiological conditions, lncRNAs preserve cartilage homeostasis, promote chondrocyte proliferation, inhibit chondrocyte apoptosis, and induce cartilage regeneration [225,226,227]. During the onset and development of OA, the expression of lncRNAs may be dysregulated, thus participating in the pathogenesis and progression of OA [228]. This is supported by the fact that the lncRNA alteration can increase the expression of MMP3, MMP9, ADAMTS5, bone morphogenic protein 2, and VEGF, resulting in degradation, hypertrophic remodeling, and vascular invasion of cartilage [229,230]. The downregulation of LncRNA maternally expressed gene 3 (MEG3) in human OA cartilage is associated with an increase in VEGF, which promotes angiogenesis and contributes to progressive joint damage [231]. Furthermore, reduced expression of MEG3 is involved via the miRNA16/SMAD7 axis in OA progression in an OA rat model [232]. Furthermore, it is associated with high levels of miRNA-34a; decreased Klotho, FGF 23, and Bcl2; and increased Bax, transforming growth factor beta 1, and caspases in LPS-stimulated C28/I2 cells. This reduces mineralization and increases ECM degradation and apoptosis [233].

### 5.1. MALAT1 and HOXA11-AS

**Figure 5:**
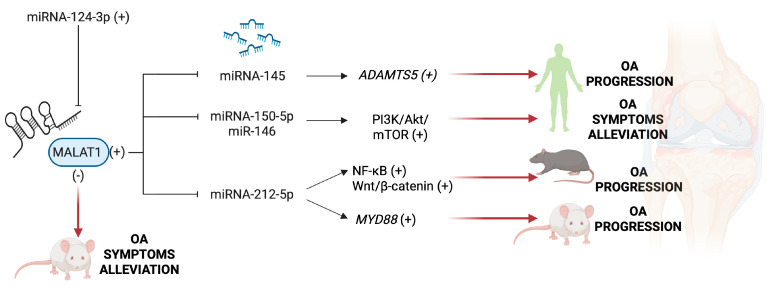
Effects of MALAT1 upregulation in OA. Abbreviations: NF-κB, nuclear factor kappa B; ADAMTS5, disintegrin and metalloproteinase with thrombospondin motifs 5; PI3K/Akt/mTOR, phosphatidylinositol 3-kinase/protein kinase B/mammalian target of rapamycin; MyD88, myeloid differentiation primary response 88. (−) Downregulated. (+) Upregulated. Image created by BioRender (https://app.biorender.com, accessed on 30 June 2025).

The upregulation of lncRNA metastasis-associated lung adenocarcinoma transcript 1 (MALAT1) increases ADAMTS5 expression and cartilage destruction by miRNA-145 downregulation in human OA cartilage (Figure 5) [234]. Suppression of MALAT1 attenuates OA progression by downregulating MyD88 protein levels through upregulation of miRNA-212-5p in the rat model [235] and by reducing NF-κB and Wnt signaling in OA mouse chondrocytes [236]. It has also been shown that overexpression of miRNA124-3p may mediate the reduction of cartilage damage in OA by impairing the stability of MALAT1 [237]. Other data from the literature suggest that MALAT1 overexpression promotes proliferation and inhibits apoptosis, inflammation, and cartilage destruction by decreasing the expression of miRNAs 150-5p and miRNA-146a, leading to an increase in Akt3 expression and activation of the PI3K/Akt/mTOR signaling pathway [238,239]. Activation of the PI3K/Akt/mTOR pathway, decreased apoptosis, and increased proliferation of chondrocytes have also been attributed to high levels of lncRNA homeobox (HOX) A11 antisense (HOXA11-AS) and low levels of miRNA-506-3p found in human OA cartilage and chondrocytes (Figure 5) [240].

### 5.2. HOTAIR and PACER

**Figure 6:**
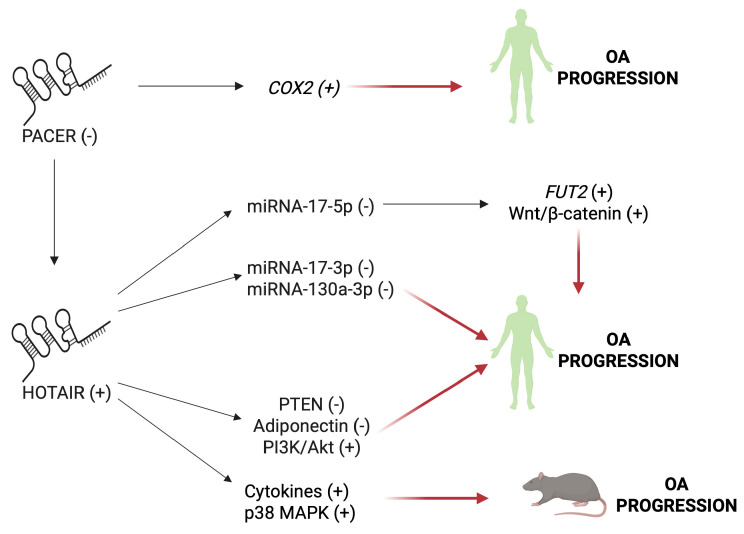
Effects of deregulating HOTAIR and PACER in OA. Abbreviations: COX2, cyclossigenase-2; FUT2, α-1,2-fucosyltransferase; PI3K/Akt, phosphatidylinositol-3-kinase/protein kinase B; p38 MAPK, p38 mitogen-activated protein kinase; PTEN, phosphatase and tensin homolog of chromosome 10. (−) Downregulated. (+) Upregulated. Image created by BioRender (https://app.biorender.com, accessed on 30 June 2025).

Overexpression of HOX transcript antisense RNA (HOTAIR), another major lncRNA involved in OA progression, causes inflammation, cell damage, and apoptosis by reducing miRNA-17-3p in LPS-stimulated C28/I2 chondrocytes [241], and miRNA-130a-3p in human OA cartilage [242]. High levels of HOTAIR promote cartilage degradation through the downregulation of miRNA-17-5p, the increase in α-1,2-fucosyltransferase 2 (FUT2), and the downregulation of the expression of Wnt inhibitory factor 1, with consequent activation of the Wnt/β-catenin pathway (Figure 6) [243,244]. The HOTAIR upregulation has been found also in OA human peripheral blood mononuclear cells (PBMCs) and promotes the inflammation response by reducing the expression of PTEN and adiponectin, and increasing the phosphorylation and subsequent activation of PI3K/Akt in human OA chondrocytes [245]. In the rat knee OA model, the elevation of the lncRNA HOTAIR upregulates serum IL-1β and TNFα levels, and p38 MAPK expression and phosphorylation in tissue cartilage, resulting in cartilage inflammation and ECM degradation [246]. HOTAIR expression is regulated by the lncRNA, p50-associated cyclooxygenase 2-extragenic RNA (PACER), whose levels are inversely related to those of HOTAIR [247]. In fact, decreased PACER expression is related to OA pathogenesis by inducing inflammation through the upregulation of COX/PGE2 levels (Figure 6) [248].

### 5.3. IGFBP7-OT, ELDR, MCM3AP-ASI

The lncRNA ENST00000512512.1, called human insulin-like growth factor binding protein-7-OT (IGFBP7-OT), and the lncRNA EGFR long non-coding downstream RNA (ELDR) are overexpressed in human OA cartilage and chondrocytes [249,250]. Upregulation of IGFBP7-OT accelerates OA progression by reducing methylation of the IGFBP7 promoter, resulting in the overexpression of IGFBP7, apoptosis, and reduced chondrocyte viability [249]. High levels of ELDR promote chondrocyte senescence and cartilage degradation by increasing the expression of the Indian hedgehog signaling molecule, involved in OA progression, by regulating histone modifications of the promoter region [250]. In OA, the overexpressed lncRNA minichromosome maintenance complex component 3 associated protein antisense 1 (MCM3AP-AS1) is involved in cell apoptosis and ECM degradation by inhibiting the expression of miRNA-149-5p with consequent activation of Notch1 [251].

### 5.4. PVT1 and GAS5

The LncRNA plasmacytoma variant translocation 1 (PVT1) overexpression reduces proliferation and increases apoptosis and ECM degradation through the downregulation of miRNA-497 and consequent activation of Akt3 [252]. High levels of PVT1 are associated with low levels of the lncRNA and growth arrest-specific 5 (GAS5), and this combination promotes LPS-induced apoptosis in OA chondrocytes [253]. However, GAS5 has been shown to be upregulated in human OA serum, cartilage, and chondrocytes, promoting apoptosis and inhibiting proliferation by downregulating miRNA-137 [254]. In addition, the upregulation of GAS5 leads to ECM degradation, inflammation, and apoptosis, but in contrast, it increases the anti-apoptotic factor Bcl2 through the downregulation of miRNA-34a [255].

### 5.5. NEAT1 and LE

High levels of the lncRNA nuclear enriched abundant transcript 1 (NEAT1) promote apoptosis, inflammation, cartilage degradation, and cell death through the downregulation of miRNA-181c and the decrease in miRNA-543 levels associated with the upregulation of phospholipase A2 group IVA expression involved in OA development [256,257]. The increase in NEAT1 expression is associated with an increase in miRNA-377-3p and stimulation of endoplasmic reticulum stress in cartilage of the OA mouse model [258], and with an upregulation of miRNA-378 in LPS-induced rat OA chondrocytes [259]. However, contrasting data show that in human OA low levels of NEAT1 are associated with upregulation of miRNA-374b-5p, leading to apoptosis and production of pro-inflammatory mediators through downregulation of post-GPI attachment to protein 1 (PGAP1) expression [260]. The decrease in the lncRNA LEMD1 antisense RNA 1 (LEMD1-AS1) also causes the downregulation of PGAP1 expression, but through the increase in miRNA-944 expression, promoting apoptosis and inflammation in OA [261].

### 5.6. SNHG Family

Dysregulated expression of lncRNAs belonging to the small nucleolar RNA host gene (SNHG) family may be associated with the progression and development of OA. For example, SNHG12, SNHG14, and SNHG16 are overexpressed in human OA cartilage and promote OA progression by reducing chondrocyte viability and increasing inflammatory response, apoptosis, and ECM degradation through the inhibition of miRNA-16-5p, miRNA-137, and miRNA-373-3p, respectively [262,263,264]. Reduced expression of SNHG1, SNHG5, SNHG7, and SNHG15 affects chondrocyte viability, proliferation, apoptosis, and migration in human OA cartilage. In particular, low levels of SNHG1 induce metabolic dysfunction by upregulating the levels of MMPs, ADAMTS4/5, and pro-inflammatory mediators, and downregulating collagen II and aggrecan through the activation of miRNA-16-5p-mediated p38 MAPK and NF-κB pathways [265]. Downregulation of SNHG5 is associated with high levels of miRNA-26a and low levels of SOX2, with a consequent reduction in chondrocyte proliferation and migration [266]. Furthermore, low levels of SNHG5 promote apoptosis by increasing miRNA-10-5p and decreasing histone H3 family 3B [267], and promote apoptosis, inhibition of viability, and increase in ADAMTS5 and MMP-13 levels by upregulating miRNA-181a-5p and downregulating transforming growth factor beta receptor 3 [268]. Downregulation of SNHG7 A causes inflammation, apoptosis, and antiproliferative effects through upregulation of miRNA-34a-5p and miRNA-214-5p, and downregulation of the synovial apoptosis inhibitor 1 and peroxisome proliferator-activated receptor gamma coactivator 1-beta pathway, respectively [269,270]. In addition, the low levels of SNHG7 contribute to inflammation and apoptosis through the overexpression of miRNA-324-3p, the downregulation of dual-specificity phosphatase 1, and the consequent activation of the p38 MAPK signaling pathway [271]. Low levels of SNGH15 induce apoptosis and ECM degradation while reducing proliferation by increasing the expression of miRNA-141-3p and miRNA-7. This results in reduced expression of Bcl2-like protein 13 and Krüppel-like factor 4, respectively [272,273]. Low levels of SNHG9 and high levels of miRNA- in OA synovial fluid and chondrocytes lead to increased apoptosis [274].

### 5.7. Other lncRNAs

In human OA synovial tissue and fibroblasts, LncRNA X-inactive specific transcript (Xist) is overexpressed, together with downregulation of miRNA-150-5p and upregulation of vascular cell adhesion protein 1 and the monocyte marker CD11b, suggesting high monocyte infiltration [275]. Xist is also associated with human OA progression by increasing FUT1 expression through binding to TATA-box binding protein-associated factor 15 [276].

The overexpression of the lncRNA TM1-3Pcauses inflammation and ECM degradation through downregulation of miRNA-144-3p and subsequent upregulation of the transcription factor one cut homeobox 2 [277].

The literature on lncRNA H19 and the lncRNA KCNQ1 overlapping transcript 1, also known as KCNQ1OT1, is conflicting, with studies linking their role in human OA progression to both up- and downregulation [278,279,280,281,282]. Specifically, overexpression of H19 decreases miRNA-130 and miRNA-106a-5p levels, promoting apoptosis and inflammation processes [278,279]. On the contrary, low levels of H19 increase miRNA-106b-5p expression and decrease tissue inhibitor of metalloproteinases 2, causing cartilage damage and decreasing proliferation and migration of chondrocytes [280]. Upregulation of KCNQ1OT1 causes inflammation and ECM degradation by decreasing miRNA-211-5p and increasing transcription factor 4 expression in cartilage [281]. It also inhibits miRNA-1202 and increases the expression of E26 transcription factor-1, which regulates many cartilage genes, such as those of MMPs in the serum of OA patients [282]. Conversely, KCNQ1OT1 downregulation A promotes ECM degradation by increasing miRNA-126-5p and subsequently decreasing the transcriptional repressor GATA binding 1 [283].

Upregulation of lncRNA LINC01094, lncRNA actin filament-associated protein 1 (AFAP1-AS1), and lncRNA LINC00958 occurs in human OA cartilage. Their role in ECM degradation, inflammation, and apoptosis is due to a respective decrease in miR-577, miR-512-3p, and miR-214-3p. This leads to an increase in the expression of the metal-regulatory transcription factor 1 (MTF-1), MMP-13, and forkhead box M1 (FOXM1) [284,285,286].

Increased levels of the lncRNA bladder cancer-associated transcript 1 (BLACAT1) promote apoptosis and ECM degradation through the downregulation of miRNA-149-5p and subsequent activation of 3-hydroxy-3-methylglutaryl-CoA reductase in IL-1β-stimulated human chondrocytes [287].

The LncRNA OPA interacting protein 5 antisense RNA 1 (OIP5-AS1) is downregulated in human OA, and its apoptotic, antiproliferative, and inflammatory role occurs through overexpression of miRNA-338-3p [288], and through overexpression of miRNA-29b-3p and downregulation of progranulin via activation of the PI3K/AKT pathway [289].

The downregulation of lncRNA WDR11 divergent transcript (lncRNA WDR11-AS1) d in human OA is associated with increased expression of the polyadenylate binding protein 1 and ECM degradation [290], whereas low levels of the lncRNA PMS2L2 cause upregulation of miRNA-34a expression, resulting in reduced chondrocyte proliferation [291].

In cartilage and synovial tissue of the OA rat model and in IL-1β-stimulated mouse chondrocyte-like ADTC5 cells, the lncRNA colorectal neoplasia differentially expressed (CRNDE) is downregulated, causing cartilage damage, inflammation, and apoptosis by decreasing the expression of dapper antagonist of catenin-1 [292].

Table 3 summarizes the effects and targets of major differentially expressed lncRNAs associated with human OA.

## 6. Cross-Talk Between Oxidative Stress and lncRNAs in Osteoarthritis

An association between altered lncRNA expression and oxidative stress by modulating transcription factors has been demonstrated in several pathologies [46]. Some lncRNAs are able to affect the antioxidant transcription factor Nrf2 signaling pathway [293,294], and it has been shown that decreased expression of lncRNA NR024118 is associated with oxidative stress, apoptosis, and inflammation by activating NF-κB expression levels and inhibiting Nrf2 signaling pathways in LPS-treated ATDC5 chondrocytes. In fact, in these cells, NR024118 overexpression attenuates the production of ROS and inflammatory cytokines, and inhibits and promotes the activation of NF-κB and Nrf2 pathways, respectively [295].

Reduced expression of the lncRNA zinc finger NFX1-type containing 1 antisense 1 (ZFAS1) and NEAT1_2in OA increases ROS levels, and decreases the activity of the antioxidant enzymes, SOD and catalase, causing apoptosis, inflammation, and ECM degradation in OA cell models. However, ZFAS1 mediates these effects by upregulating miRNA-1323 levels [208,296]. The increase in HOTAIR expression promotes oxidative stress, as evidenced by high levels of ROS and malondialdehyde and low levels of SOD activity, and causes the production of pro-inflammatory factors, MMPs, and ADAM10 through the downregulation of miRNA-222-3p in IL-1β-treated C28/I2 chondrocytes [184].

In human OA chondrocytes, high levels of the lncRNA CIR are accompanied by downregulation of miRNA-130a, upregulation of B-cell lymphoma 2 interacting mediators of cell death, and accumulation of ROS. Furthermore, high ROS levels increase the expression of lncRNA CIR, suggesting a reciprocal interaction between this lncRNA and oxidative stress [297].

Knockdown of SNHG1 increases the production of ROS, while H_2_O_2_ downregulates SNHG1 expression and upregulates levels of miRNA-195. This induces apoptosis and an inflammatory state in chondrocytes through NF-κB activation and the production of pro-inflammatory mediators [183].

The lncRNA LOC727924, also known as LINC02203, is upregulated in human OA cartilage and chondrocytes; and increases intracellular ROS, apoptosis, inflammation, and ECM degradation through the downregulation of miRNA-26a and the consequent upregulation of KPNA3. The immunosuppressive and anti-inflammatory vasoactive intestinal peptide reduces LOC727924 levels and the accumulation of ROS, in addition to improving chondrocyte function [189].

In the synovial fluid of OA patients, the downregulation of MEG3 is involved in ferroptosis by increasing levels of miRNA-885-5p, which results in decreased SLC7A11 and GPX4. Conversely, the overexpression of this lncRNA reduces erastin-induced ferroptosis by downregulating miRNA-885-5p and upregulating SLC7A11 and GPX4 [298].

Overexpression of GAS5 in OA fibroblast-like synoviocytes (FLSs) is associated with reduced expression of SLC7A11, GPX4, Nrf2, and HO-1, as well as low activity of SOD. It is also accompanied by increased levels of long-chain fatty acid CoA ligase 4, an important enzyme involved in the ferroptosis pathway, ROS, and p53. This implies a reduced viability and antioxidant capacity, leading to cell death, oxidative stress, inflammation, and ferroptosis, which are mediated by the downregulation of miRNA-205 [299]. Decreased SNHG7 is also associated with ferroptosis through an increase in miRNA-485-5p, resulting in ROS production and the downregulation of ferroptosis suppressor protein 1, a glutathione-independent ferroptosis inhibitor molecule [300].

The targets and effects of the major lncRNAs related to oxidative stress in OA are shown in Table 4.

## 7. Conclusions

OA causes joint degeneration, pain, and reduced physical activity, thus negatively impacting quality of life. This review reports on the main mechanisms involved in the role of oxidative stress, as well as the most important dysregulated miRNAs and lncRNAs in the pathogenesis of OA. Furthermore, the cross-talk between oxidative stress and these ncRNAs has been discussed. In particular, the dysregulation of several miRNAs and lncRNAs has been shown to be associated with ROS production, and oxidative stress may be responsible for the altered expression of these ncRNAs. Importantly, many oxidative stress-related lncRNAs interact with miRNAs, making the role of oxidative stress in OA pathogenesis more complex. The interplay between miRNAs and lncRNA plays a crucial role in the pathogenesis of OA, influencing key processes such as cartilage degradation, synovial inflammation, and chondrocyte apoptosis. There is emerging evidence that specific miRNAs and lncRNAs, which are modulated by oxidative stress and vice versa, are important regulators of signaling pathways involved in joint homeostasis, offering promising therapeutic implications. Thus, identifying the molecular mechanisms and pathways involved in the intricate interaction between oxidative stress, miRNAs, and lncRNAs in OA may help to identify therapeutic targets to restore the normal expression of these ncRNAs by inhibiting oxidative stress, or to restore the physiological redox state by normalizing the levels of miRNAs and lncRNAs. In this context, recent experimental findings have shown the promising application of anti-sense oligonucleotides, named anti-miRNA oligonucleotides (AMOs) and anti-lncRNAs (ASOs), to suppress altered miRNA and lncRNA expression in several human pathologies [301,302], suggesting that the development of targeted AMO- and ASO-based approaches could be applicable in the future for miRNAs and lncRNAs involved in the onset of oxidative stress in OA. On the contrary, the use of molecules with antioxidant properties could regulate the intracellular redox state and consequently normalize the levels of redox-regulated miRNAs and lncRNA. Indeed, several antioxidants, such as curcumin, resveratrol, and quercetin, affect the expression of specific miRNA and lncRNA in cancer [303,304,305]. Additionally, the levels of many ncRNAs in biological fluids are attracting increasing attention as potential biomarkers for various diseases [306]. The evaluation in OA of the expression pattern of miRNAs and lncRNAs, which are related to oxidative stress, and whose molecular mechanisms are known, could help to direct patients toward personalized approaches for the prevention and treatment of the disease. In this sense, it is desirable in the future to stimulate the development of drugs that can inhibit the loss of cartilage homeostasis and/or reduce the progression of osteoarthritic pathology.

## Figures and Tables

**Table 1:** Major differentially expressed miRNAs in human OA and their major targets and effects.

miRNA	Targets	Effects	Human Model/Cell Type	Ref.
miRNA-130a (−)	TNFα (+)	Inflammation	OA chondrocytes	[148]
miRNA-149 (−)	TAK1/NF-κB (+) VCAM-1 (+) p-Akt (+)	Inflammation Apoptosis Inflammation	OA chondrocytes OA cartilage	[149] [152]
miRNA-373 (−)	P2X7R (+)	Inflammation Proliferation (−)	OA chondrocytes	[150]
miRNA-24-3p (−)	BCL2L12 (+)	Inflammation ECM degradation	IL-1β-stimulated CHON-001 cells	[151]
miRNA-92a-3p	NF-κB (+) MAPKs (+)	ECM degradation	IL-1β-stimulated chondrocytes	[153]
miRNA-497-5p (−)	Wnt/β-catenin (+)	ECM degradation	IL-1β-stimulated chondrocytes	[154]
miRNA-128-3p	WISP1 (+)	Inflammation ECM degradation Apoptosis Proliferation (−)	OA cartilage IL-1β-stimulated C28/I2 cells	[155]
miRNA-382-3p (−)	CX43 (+) TLR4/MyD88/ NF-κB (+)	Inflammation ECM degradation	IL-1β-stimulated NHAC-kn	[156]
miRNA-195 (+)	PTHrP (−)	ECM degradation	OA cartilage	[157]
miRNA-140 (−)	MMP3 (+)	ECM degradation	OA synovial fluid	[158]
miRNA-190 (−)	MMP3 (+)	ECM degradation	OA synovial fluid	[158]
miRNA-548d-5p (−)	SP1 (+)	Inflammation ECM degradation Apoptosis Proliferation (−)	IL-1β-stimulated C28/I2 cells	[159]
miRNA-381a-3p (+)	IkBα (−) NF-κB (+)	Inflammation ECM degradation	OA cartilage and synovium	[160]
miRNA-454 (+)	Stanniocalcin-1 (−) NF-κB (+)	ECM degradation	OA Synovial Fibroblast-like cells	[161]
miRNA-760 (+)	HBEGF (−)	ECM degradation	OA cartilage	[162]
miRNA-145 (−)	MKK4 (+)	ECM degradation	OA cartilage	[163]
miRNA-146a-5p (+)	NF-κB (+)	Inflammation ECM degradation	OA cartilage	[164]
miRNA-122 (+)	Sirt1 (−)	ECM degradation	OA cartilage	[165]
miRNA-140-5p (−)	Jagged1/Notch (+)	Differentiation alteration Apoptosis Proliferation (−)	CPSC from OA cartilage	[166]
miRNA-320a (−)	DAZAP1 (+) MAPKs (+)	Inflammation Apoptosis Proliferation (−)	IL-1β-stimulated HC-A cells	[167]
miRNA-146a (+)	VEGF (+) Smad4 (−)	Apoptosis	Mechanically stressed chondrocytes	[168]
miRNA-379-5p (−)	YBX1 (+) PI3K/Akt (+)	Proliferation (−) Expression of ECM proteins (−)	OA cartilage	[169]
miRNA-99a (−)	Frizzled 8 (+)	ECM degradation Apoptosis	Cytokine-stimulated SW1353 cells	[170]
miRNA-33b-3p (−)	DNMT3A (+)	ECM degradation Apoptosis Proliferation (−)	OA cartilage IL-1β-stimulated chondrocytes	[171]
miRNA-182-5p (+)	FGF9 (−)	ECM degradation Apoptosis Proliferation (−)	OA chondrocytes	[172]
miRNA-34a (+)	Sirt1 (−)	ECM degradation Apoptosis Proliferation (−)	OA chondrocytes	[173]

Abbreviations: TAK1, transforming growth factor-β activated kinase 1; NF-κB, nuclear factor kappa B; VCAM-1, vascular cell adhesion molecule-1; p-Akt, phosphoprotein kinase B; TNFα, tumor necrosis factor α; MMP, metalloproteinase; IL-1β, interleukin-1β; P2X7R, purinergic P2X7 receptor; BCL2L12, B-cell leukemia 2-like 12; SP1, specificity protein 1; IkBα, inhibitor kappa B-alpha; HBEGF, heparin-binding EGF-like growth factor; MKK4, mitogen-activated protein kinase kinase 4; VEGF, vascular endothelial growth factor; DAZAP1, DAZ-associated protein 1; MAPKs, mitogen-activated protein kinases; CPSC, cartilage progenitor/stem cells; YBX1, Y-box binding protein 1; PI3K/Akt, phosphatidylinositol-3-kinase/protein kinase B; CX43, Connexin 43; TLR4/MyD88/NF-κB, Toll-like receptor 4/Myeloid differentiation primary response 88/NF-κB; NHAC-kn, normal human articular chondrocytes-knee; FGF9, Fibroblast growth factor 9; Sirt1, sirtuin-1; DNMT3A, DNA methyltransferase 3A; PTHrP, parathyroid hormone-related protein; ECM, extracellular matrix; WISP1, Wnt-1 inducible signaling pathway protein 1. (−) Downregulated. (+) Upregulated.

**Table 2:** Main miRNAs involved in the cross-talk with oxidative stress in OA.

miRNA	Targets	Effects	Model/Cell Type	Ref.
miRNA-93-5p (−)	MAP3K8 (+) iNOS (+) COX2 (+)	Inflammation ECM degradation Oxidative stress	Human chondrocytes treated with H_2_O_2_ and stimulated with IL-1β	[180]
miRNA-34a-5p (+)	Sirt1/p53 (−)	Inflammation Apoptosis	tBHP-treated HC-OA cells	[181]
miRNA-9 (+)	Sirt1 (−)	Cartilage damage	H_2_O_2_-treated human chondrocytes H_2_O_2_-treated C28/I2 cells	[182]
miRNA-195 (+)	IKKα (−) NF-κB (+)	Apoptosis	H_2_O_2_-treated C28/I2 cells	[183]
miRNA-222-3p (−)	ADAM10 (+)	Inflammation ECM degradation Apoptosis Oxidative stress	IL-1β-stimulated C28/I2 cells	[184]
miRNA-486-5p (+)	Nrf1 (−)	Oxidative stress Apoptosis Inflammation	LPS-stimulated ATDC5 cells	[185]
miRNA-505-3p (+)	Sirt3 (−)	Inflammation Oxidative stress Apoptosis ECM degradation	Human OA cartilage	[186]
miRNA-26a (−) miRNA-26b (−)	KPNA3 (+) NF-κB (+)	Oxidative stress ECM degradation	Human OA cartilage and chondrocytes	[188] [189]
miRNA-485-3p (−)	Notch2 (+) NF-κB (+)	Oxidative stress Inflammation ECM degradation Apoptosis	Human OA cartilage LPS-stimulated SW1353 and CHON-001 cells	[190]
miRNA-27 (−)	TLR4 (+) NF-κB (+)	Oxidative stress ECM degradation	IL-1β-stimulated chondrocytes	[192]
miRNA-127-5p (−)	TLR4 (+)	Oxidative stress Inflammation ECM degradation Apoptosis	Human OA cartilage	[193]
miRNA-24-3p (−)	CTSB (+)	Oxidative stress Apoptosis Inflammation	Human OA cartilage IL-1β-stimulated CHON-001 cells	[194]
miRNA-197-3p (−)	SOX5 (+)	Oxidative stress Inflammation ECM degradation Apoptosis	Human OA cartilage IL-1β-stimulated C28/I2 cells	[195]
miRNA-203-3p (−)	MYD88/NF-κB (+)	Oxidative stress Apoptosis ECM degradation Pyroptosis	In vitro and in vivo rat models of OA	[197]
miRNA-181-5p (+)	SBP2 (−) GPX (−)	Oxidative stress Cartilage damage	Human OA chondrocytes IL-1β-stimulated SW1353 cells	[198]
miRNA-375 (+)	JAK2/STAT3 (−)	Oxidative stress Apoptosis ECM degradation	Mouse OA models	[199]
miRNA-34a (+) miRNA-146a (+) miRNA-181a (+)	NF-κB (+) Sirt1 (−) Nrf2 (+) SOD2 (+) Wnt/β-catenin (+)	Inflammation Apoptosis Proliferation (−) Oxidative stress Oxidative stress Apoptosis ECM degradation	Adipokines-stimulated human OA synoviocytes HP-stimulated human OA chondrocytes	[201] [209]
miRNA-34a (+) miRNA-181a (+)	NF-κB (+)	Oxidative stress Apoptosis	Visfatin-stimulated human OA chondrocytes	[202]
miRNA-34a (+) miRNA-146a (−)	Nrf2 (+) SOD2 (+) CAT (+) GPX (+)	Apoptosis Cell viability (−)	H_2_O_2_-treated human chondrocytes	[203]
miRNA-146a (+)	Nrf2/HO-1 (−)	Oxidative stress ECM degradation	Rat OA chondrocytes	[207]
miRNA-1323 (+)	Nrf2/OH1 (−)	Inflammation Apoptosis Oxidative stress	LPS-stimulated human OA chondrocytes	[208]
miRNA-181b (+)	SLC7A11 (−)	Oxidative stress Ferroptosis	Human and rat chondrocyte models of OA	[210]
miRNA-1 (−)	CX43 (+) SLC7A11 (−) GPX (−)	Oxidative stress Ferroptosis	Human OA cartilage and IL-1β-stimulated human chondrocytes	[211]

Abbreviations: MAP3K8, mitogen-activated protein kinases kinase kinase; iNOS, inducible nitric oxide synthase; COX2, cyclooxygenase; Sirt, sirtuin; tBHP, tert-butyl hydroperoxide; IKKα, inhibitory kappa B kinase α; NF-κB, nuclear factor kappa B; ADAM10, disintegrin and metalloprotease domain-containing protein 10; Nrf2/HO-1, nuclear factor erythroid 2-related factor 2/heme oxygenase-1; SOD, superoxide dismutase; CAT, catalase; HP, hydrostatic pressure; KPNA3, karyopherin subunit alpha 3; TLR4, Toll-like receptor 4; CTSB, cathepsin B; SOX5, SRY-box transcription factor 5; SBP2, selenocysteine insertion sequence-binding protein 2; GPX, glutathione peroxidase; SLC7A11, ferroptosis protective protein, solute carrier family 7 member 11; CX43, connexin 43; JAK2/STAT3, Janus kinase 2/signal transducer and activator of transduction; MYD88, myeloid differentiation primary response 88; LPS, lipopolysaccharide; IL-1β, interleukin-1β; ECM, extracellular matrix. (−) Downregulated. (+) Upregulated.

**Table 3:** Major differentially expressed lncRNAs in human osteoarthritis (OA) and their major targets and effects.

lncRNA	Targets	Effects	Model/Cell Type	Ref.
MEG3 (−)	VEGF (+) miRNA34a (+) Kloto (−), FGF23 (−), Bcl2 (−), Bax (+), caspases (+)	Angiogenesis Mineralization (−) ECM degradation Inflammation Apoptosis	OA cartilage LPS-stimulated C28/I2 cells	[231] [233]
MALAT1 (+)	miRNA-145 (−) ADAMTS5 (+) miRNA-150-5p (−) AKT3 (+)	ECM degradation Proliferation Apoptosis (−) ECM degradation (−)	OA cartilage IL-1β-stimulated chondrocytes OA cartilage IL-1β-stimulated chondrocytes	[234] [238]
	miRNA-146a (−) PI3K/AKT/mTOR (+)	Inflammation (−) Apoptosis (−)	LPS-stimulated chondrocytes	[239]
HOXA11-AS (+)	miRNA-506-3p (−) PI3K/AKT/mTOR (+)	Proliferation Apoptosis (−)	OA cartilage and chondrocytes	[240]
HOTAIR (+)	miRNA-17-3p (−) miRNA-130a-3p (−) miRNA-17-5p (−) FUT2 (+), WIF-1 (−), Wnt/β-catenin (+) Adiponectin (−) PTEN (−), PI3K/AKT (+)	Inflammation Apoptosis Cell damage ECM degradation Inflammation	LPS-stimulated C28/I2 cells OA cartilage and chondrocytes IL-1β-stimulated chondrocytes OA chondrocytes OA PBMC and chondrocytes	[241] [242] [243] [244] [245]
PACER (−)	HOTAIR (+)	Inflammation	OA blood and OA cell model	[247] [248]
IGFBP7-OT (+)	*IGFBT* promoter methylation (−)	Inflammation Apoptosis Cell viability (−)	OA cartilage and chondrocytes	[249]
ELDR (+)	Indian hedgehog signaling molecule (+)	ECM degradation Chondrocyte senescence	OA cartilage and chondrocytes	[250]
MCM3AP-AS1 (+)	miRNA-149-5p (−) Notch1 (+)	ECM degradation Apoptosis	OA cartilage IL-1β-stimulated chondrocytes	[251]
PVT1 (+)	GAS5 (−)	Apoptosis	OA synovial fluid LPS-stimulated HC-OA cells	[253]
GAS5 (+)	miRNA-137 (−) miRNA-34a (−) Bcl2 (+)	Apoptosis Proliferation (−) ECM degradation Inflammation Apoptosis	OA serum, cartilage and chondrocytes OA chondrocytes	[254] [255]
NEAT1 (+) NEAT1 (−)	miRNA-181c (−) miRNA-543 (−), PLA2G4A (+) miRNA-374b-5p (+) PGAP1 (−)	Inflammation Apoptosis ECM degradation Inflammation Apoptosis	OA cartilage and chondrocytes OA cartilage LPS-stimulated chondrocytes	[256] [257] [260]
LEMD1-AS1 (−)	miRNA-944 (+) PGAP1 (−)	Inflammation Apoptosis	OA cartilage LPS-stimulated chondrocytes	[261]
SNHG12 (+)	miRNA-16-5p (−)	Inflammation Apoptosis ECM degradation	OA cartilage	[262]
SNHG14 (+)	miRNA-137 (−)	Inflammation Apoptosis ECM degradation Cell viability (−)	OA cartilage	[263]
SNHG16 (+)	miRNA-373-3p (−)	Inflammation Apoptosis ECM degradation Cell viability	OA cartilage	[264]
SNHG1 (−)	miRNA-16-5p (+) p38 MAPK/NF-κB (+)	Inflammation Apoptosis ECM degradation	IL-1β-stimulated chondrocytes	[265]
SNHG5 (−)	miRNA-26a (+) SOX2 (−) miRNA-10-5p (+) H3F3B (−) miRNA-181a-5p (+) TGFβR3 (−)	Proliferation (−) Migration (−) Apoptosis Proliferation (−) Apoptosis (+) ECM degradation	OA cartilage IL-1β-stimulated chondrocytes IL-1β-stimulated C20/A4 cells	[266] [267] [268]
SNHG7 (−)	miRNA-34a-5p (+) SYVN1 (−) miRNA-214-5p (+) PPARGC1B (−) miRNA-324-3p (+) DUSP1 (−) P38 MAPK (+)	Inflammation Apoptosis Proliferation (−) Inflammation Apoptosis Proliferation (−) Inflammation Apoptosis	IL-1β-stimulated chondrocytes IL-1β-stimulated chondrocytes IL-1β-stimulated chondrocytes	[270] [277] [271]
SNHG15 (−)	miRNA-141-3p (+) Bcl2-L-13 (−) miRNA-7 (+) KLF4 (−)	Inflammation Apoptosis Proliferation (−) Proliferation (−) Apoptosis	IL-1β-stimulated chondrocytes OA cartilage IL-1β-stimulated chondrocytes	[272] [273]
SNHG9 (−)	miRNA-34a (+)	Apoptosis	OA synovial fluid and chondrocytes	[274]
Xist (+)	miRNA-150-5p (−) VCAM1 (+) CD11b (+) FUT1 (+), TAF15 (+)	Monocyte infiltration OA progression	OA synovial tissue and fibroblasts OA cartilage	[275] [276]
TM1-3P (+)	miRNA-144-3p (−) ONECUT2 (+)	ECM degradation Inflammation	IL-1β-stimulated synovial fibroblasts	[277]
H19 (+)	miRNA-130 (−) miRNA-106a-5p (−)	Inflammation Apoptosis Inflammation Apoptosis	OA cartilage IL-1β-stimulated chondrocytes LPS-stimulated C28/I2 cells	[278] [279]
H19 (−)	miRNA-106b-5p (+) TIMP2 (−) MMP3 (+) ADAMTS5 (+)	ECM degradation Proliferation (−) Migration (−)	IL-1β-stimulated chondrocytes	[280]
KCNQ1OT1 (+) KCNQ1OT1 (−)	miRNA-211-5p (−) TCF4 (+) miRNA-1202 (−) ETS1 (+) miRNA-126-5p (+) TRPS1 (−)	Inflammation ECM degradation Inflammation ECM degradation ECM degradation	OA cartilage LPS-stimulated C28/I2 cells OA serum OA cartilage	[281] [282] [283]
LINC01094 (+)	miRNA-577 (−) MTF1 (+)	Apoptosis	OA cartilage LPS-stimulated chondrocytes	[284]
AFAP1-AS1 (−)	miRNA-512-3p (−) MMP13 (+)	ECM degradation	OA cartilage	[285]
LINC00958 (+)	miRNA-214-3p (−) FOXM1 (+)	Inflammation Apoptosis Cell viability (−)	IL-1β-stimulated CHON-001	[286]
BLACAT1 (+)	miRNA-149-5p (−) HMGCR (+)	ECM degradation Apoptosis	IL-1β-stimulated chondrocytes	[287]
OIP5-AS1 (−)	miRNA-338-3p (+) PI3K/Akt (+) miRNA29b-3p (+) progranulin (−)	Inflammation Apoptosis ECM degradation Proliferation (−) Cell viability (−)	IL-1β-stimulated chondrocytes	[288] [289]
WDR11-AS1 (−)	PABPC1 (+)	ECM degradation	OA cartilage cytokine-stimulated chondrocytes	[290]
PMS2L2	miRNA-34a (+)	Proliferation (−)	OA synovial fluid and chondrocytes	[291]

Abbreviations: VEGF, vascular endothelial growth factor; FGF23, fibroblast growth factor 23; LPS, lipopolysaccharide; ADAMTS5, disintegrin and metalloproteinase with thrombospondin motifs 5; IL-1β, interleukin-1β; PI3K/Akt/mTor, phosphatidylinositol 3-kinase/protein kinase B/mammalian target of rapamycin; FUT, α-1,2-fucosyltransferase; WIF-1, Wnt inhibitory factor 1; PTEN, phosphatase and tensin homologue of chromosome 10; HC-OA cells, human chondrocyte–osteoarthritis cells; VCAM-1, vascular cell adhesion molecule-1; TAF15, TATA-box binding protein-associated factor 15; ONECUT2, transcription factor one cut homeobox 2; TIMP2, tissue inhibitor of metalloproteinases 2; MMP, metalloproteinase; TCF4, transcription factor 4; ETS1, E26 transcription factor-1; TRPS1, transcriptional repressor GATA binding 1; MTF1, metal-regulatory transcription factor 1; PLA2G4A, phospholipase A2 group IVA; PGAP1, post-GPI attachment to protein 1; HMGCR, 3-hydroxy-3-methylglutaryl-CoA reductase; p38 MAPK, p38 mitogen-activated protein kinase; NF-κB, nuclear factor kappa B; SOX2, SRY-box transcription factor 2; H3F3B, histone H3 family 3B; TGFβR3, transforming growth factor beta receptor 3; SYVN1, synovial apoptosis inhibitor 1; PPARGC1B, peroxisome proliferator-activated receptor gamma coactivator 1-beta; DUSP1, dual-specificity phosphatase 1; Bcl2-L-13, Bcl2-like protein 13; KLF4, Krüppel-like factor 4; PABPC1, polyadenylate binding protein 1; ECM, extracellular matrix. (−) Downregulated. (+) Upregulated.

**Table 4:** Main lncRNAs involved in the cross-talk with oxidative stress in osteoarthritis (OA).

lncRNA	Targets	Effects	Model/Cell Type	Ref.
NR024118 (−)	NF-κB (+) Nrf2 (−)	Oxidative stress Inflammation Apoptosis	LPS-stimulated ATDC5 cells	[295]
ZFAS1 (−)	miRNA-1323 (+) Nrf2/HO-1 (−) SOD (−) CAT (−)	ROS production Inflammation Apoptosis	LPS-stimulated human chondrocytes	[208]
NEAT1-2 (−)	Nrf2 (−) SOD1(−) SOD2 (−) iNOS (+)	Oxidative stress Apoptosis ECM degradation	IL-1β-stimulated human chondrocytes	[296]
HOTAIR (+)	miRNA-222-3p (−) SOD (−) MMPs (+) ADAM10 (+)	ROS production Inflammation ECM degradation	IL-1β-stimulated C28/I2 cells	[184]
CIR (+)	miRNA-130a (−) Bim (+)	ROS production ECM degradation Apoptosis	Human OA chondrocytes H_2_O_2_-treated human chondrocytes	[297]
SNHG1 (−)	miRNA-195 (+) NF-κB (+)	Inflammation (+) Apoptosis (+) ROS production	H_2_O_2_-treated C28/I2 cells C28/I2 cells	[187]
LOC727924 (+)	miRNA-26a (−) KPNA3 (+)	ROS production Inflammation Apoptosis ECM degradation	Human OA chondrocytes	[189]
MEG3 (−)	miRNA-885-5p (+) SLC7A11 (−) GPX4 (−)	Ferroptosis	Synovial OA fluid	[298]
GAS5 (+)	miRNA-205 (−) SLC7A11 (−) GPX4(−) Nrf2/HO-1 (−) ACSL4 (+) P53 (+)	ROS production Cell viability (−) Inflammation Ferroptosis	IL-1β-stimulated OA FLS	[299]
SNHG7 (−)	miRNA-485-5p (+) FSP1 (−)	ROS production Ferroptosis	IL-1β-stimulated human chondrocytes	[300]

Abbreviations: Nrf2/HO-1, nuclear factor erythroid 2-related factor/hemo-oxygenase-1; ECM, extracellular matrix; LPS, lipopolysaccharide; NF-κB, nuclear factor kappa B; SOD, superoxide dismutase; CAT, catalase; ROS, reactive oxygen species; iNOS, inducible nitric oxide synthase; MMPs, metalloproteinases; ADAM10, A disintegrin and metalloproteinase domain-containing protein 10; Bim, B-cell lymphoma 2 interacting mediators of cell death; IL-1β, interleukin-1β; KPNA3, karyopherin subunit alpha 3; SLC7A11, solute carrier family 7 member 11; GPX4, selenoprotein glutathione peroxidase 4; ACSL4, long-chain fatty acid CoA ligase 4; FSP1, ferroptosis suppressor protein 1. (−) Downregulated. (+) Upregulated.
